# A Perfusion Culture System for Assessing Bone Marrow Stromal Cell Differentiation on PLGA Scaffolds for Bone Repair

**DOI:** 10.3389/fbioe.2018.00161

**Published:** 2018-11-15

**Authors:** Caroline Moser, Katie Bardsley, Alicia J. El Haj, Mauro Alini, Martin J. Stoddart, Jennifer J. Bara

**Affiliations:** ^1^AO Research Institute Davos, Davos, Switzerland; ^2^Laboratory for Translational Nutritional Biology, Department of Health Sciences and Technologies, Institute of Food Nutrition and Health, ETH Zürich, Zürich, Switzerland; ^3^Institute for Science and Technology in Medicine, Keele University, Keele, United Kingdom; ^4^Healthcare Technology Institute, Institute of Translational Medicine, University of Birmingham, Birmingham, United Kingdom; ^5^Department of Orthopaedic Surgery, Washington University, St Louis, MO, United States

**Keywords:** perfusion bioreactor, MSCs, PLGA, bone repair, tissue engineering

## Abstract

Biomaterials development for bone repair is currently hindered by the lack of physiologically relevant *in vitro* testing systems. Here we describe the novel use of a bi-directional perfusion bioreactor to support the long term culture of human bone marrow stromal cells (BMSCs) differentiated on polylactic co-glycolic acid (PLGA). Primary human BMSCs were seeded onto porous PLGA scaffolds and cultured in static vs. perfusion culture conditions for 21 days in osteogenic vs. control media. PLGA scaffolds were osteoconductive, supporting a mature osteogenic phenotype as shown by the upregulation of Runx2 and the early osteocyte marker E11. Perfusion culture enhanced the expression of osteogenic genes Osteocalcin and Osteopontin. Extracellular matrix deposition and mineralisation were spatially regulated within PLGA scaffolds in a donor dependant manner. This, together with the observed upregulation of Collagen type X suggested an environment permissive for the study of differentiation pathways associated with both intramembranous and endochondral ossification routes of bone healing. This culture system offers a platform to assess BMSC behavior on candidate biomaterials under physiologically relevant conditions. Use of this system may improve our understanding of the environmental cues orchestrating BMSC differentiation and enable fine tuning of biomaterial design as we develop tissue-engineered strategies for bone regeneration.

## Introduction

Large bone defects and non-union fracture pose a significant socioeconomic burden, with bone being the second most transplanted tissue after blood products (Campana et al., 2014). There is increasing demand for bone grafts globally due to our aging population (Cheung, 2005) and higher incidence of fractures (Burge et al., 2007; Amin et al., 2014). Current gold standards for treatment are autologous or allogeneic bone grafts. Autologous bone grafts taken from the iliac crest are osteoinductive and osteoconductive (Khan et al., 2005). Nevertheless, the size of the graft is limited (Megas, 2005) and donor site morbidity may be observed (Younger and Chapman, 1989). Decellularized allogeneic grafts confer high osteoconductivity (Finkemeier, 2002), however, pose the risk of adverse immune responses (Bostrom and Seigerman, 2005) and disease transmission (Finkemeier, 2002). Thus, focus has turned to tissue engineering strategies.

A frequently used cell source for tissue engineering bone are human bone marrow derived stromal cells (BMSCs) (Arinzeh et al., 2003; van den Dolder et al., 2003; Cinotti et al., 2004). BMSCs may be culture expanded without losing their ability to differentiate along the osteogenic lineage and are able to produce an osseous ECM (Pittenger et al., 1999). Developmentally and in tissue engineering, bone may form via two distinct pathways; intramembranous ossification, whereby BMSCs directly differentiate into osteoblasts and endochondral ossification, whereby BMSCs first form a cartilage anlagen which is remodeled and replaced by bone (Gilbert, 2016). The differentiation pathway BMSCs undergo in tissue engineering is highly dependent on the biochemical, biophysical and mechanical microenvironment. Thus, there is a need to establish a physiologically relevant *in vitro* system for testing BMSCs responsiveness to biomaterials. As it is known that cellular behavior *in vitro* may differ from *in vivo* behavior, an *in vitro* system that effectively recapitulates the *in vivo* microenvironment is desirable (Hulsart-Billström et al., 2016). The first step in achieving this is to culture cells in 3D. The nature of cell attachment precedes and influences important events for instance cell migration and differentiation (Baker and Chen, 2012). In 2D, cell-cell and cell-ECM interactions are held to a minimum (Baker and Chen, 2012). In terms of biophysical and biomechanical properties 3D culture also provides a more physiologically relevant environment compared to 2D. However, depending upon their size, culturing 3D constructs in static conditions may lead to cell necrosis in the construct center due to mass transport limitations (Muschler et al., 2004). Perfusion culture also mimics interstitial fluid flow in the lacunar and canalicular spaces of bone (Cowin et al., 1991; Weinbaum et al., 1994). Thus, by culturing constructs under perfusion, the *in vivo* environment is more closely represented and cell survival may be improved. Shear stress generated from fluid flow is also an important driver of osteoprogenitor differentiation and bone cell activity. Perfusion culture of osteoblastic cells increases alkaline phosphatase activity, Osteopontin secretion and matrix mineralization (Bancroft et al., 2002). Likewise, shear stress generated from fluid flow is known to promote the osteogenic differentiation of BMSCs (McCoy and O'Brien, 2010; Yourek et al., 2010; Yeatts et al., 2012). In this study we cultured BMSCs on poly(lactic-co-glycolic acid) (PLGA), which fulfills many criteria for tissue engineering bone. PLGA is biocompatible, FDA approved and it is possible to modify surface properties to achieve better biocompatibility (Danhier et al., 2012). By using different scaffold engineering strategies PLGA allows for diverse macro- and microstructures. Further, PLGA is biodegradable (Gentile et al., 2014) which is beneficial in order to avoid additional surgery that would be otherwise necessary for removal.

The aim of the present study was to develop an *in vitro* biomaterials testing platform to study the osteogenic differentiation of primary human BMSCs. The influence of perfusion culture on BMSC differentiation was investigated by culturing cells on PLGA scaffolds in a closed system, bidirectional flow perfusion bioreactor.

## Materials and methods

### Bone marrow stromal cell isolation and *in vitro* expansion

Primary BMSCs were isolated from vertebral body bone marrow aspirates acquired with informed consent and full ethical approval (KEK Bern 126/03). For the experiments four donors were used (female 22 years, female 29 years, male 44 years, male 56 years). BMSCs were isolated using Histopaque-1077 (Sigma Aldrich®, Switzerland) and density centrifugation as previously described (Bara et al., 2015). Briefly, after centrifugation the mononuclear cells present in the interphase were isolated and counted using a Scepter™ handheld automated cell counter. Cells were seeded in at a density of ~50,000 cells/cm^2^ in growth media [alpha MEM (Gibco® Switzerland), 10% fetal calf serum (SeraPlus, Germany), 1% penicillin and streptomycin (Gibco®, Switzerland), 5 ng/ml basic fibroblastic growth factor (Fitzgerald Industries International, USA)] and cultured at 37°C and 5% CO_2_. After 4 days, growth media was changed 3× per week. BMSCs were cryopreserved at p1 and stored in liquid nitrogen prior to use.

### PLGA 50:50 scaffolds

**Figure 1:**
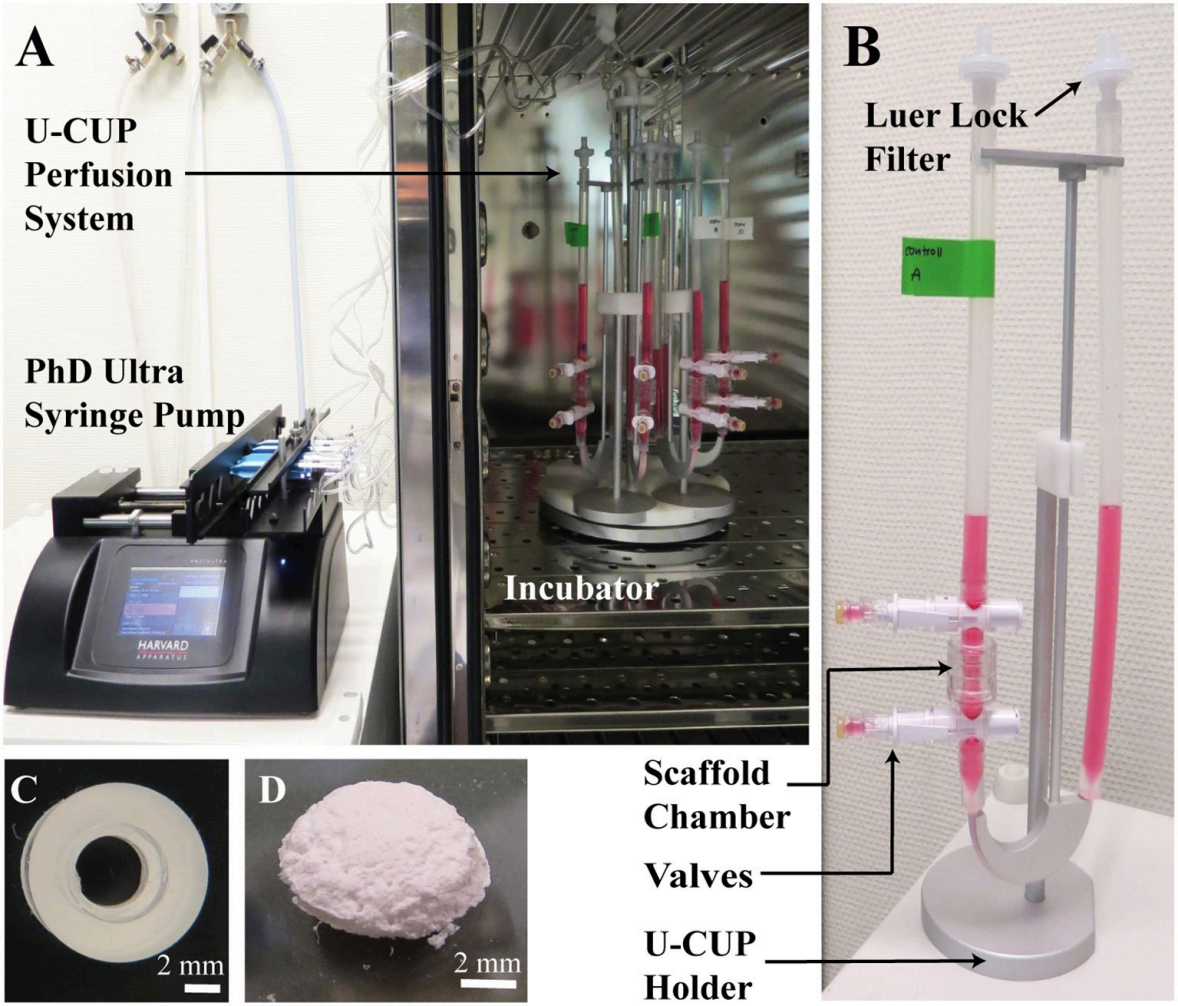
**(A,B)** The U-CUP perfusion system. The bidirectional perfusion flow system U-CUP (CELLEC BIOTEK AG, Switzerland) was used to culture PLGA scaffolds under perfusion. The PHD Ultra™ syringe pump (Havard apparatus, United States) created an oscillating flow of media in the U-CUP by pumping air back and forth. Perfusion velocity for the first 24 h was 400 μm/s, thereafter the velocity was reduced to 100 μm/s. The syringes were connected to the U-CUPs via a 22 μm filter to prevent microbes from entering the system. The holder system holding maximum 10 U-CUPs, was placed in the incubator while the syringe pump was located outside. Two scaffolds per U-CUP were positioned between two silicon scaffold holders in the perfusion scaffold chamber. Each U-CUP was filled with 10 ml of culture media. Media changes were performed via the valves top and below the scaffold chamber. **(C)** Silicon scaffold holder. Representative picture of a silicon scaffold holder; scale bar = 2 mm. **(D)** PLGA Scaffold. Representative picture of a PLGA 50:50 scaffold, the scaffolds were 8 mm in diameter, 2 mm height with a pore size of 100–150 μm; scale bar = 2 mm.

The poly(lactic-co-glycolic acid) scaffolds with a 50:50 ratio lactic acid vs. glycolic acid (PLGA 50:50) were produced as previously described (Bardsley et al., 2016). The scaffolds were 8 mm in diameter, 2 mm height with a pore size of 100–150 μm (Figure 1D). Scaffold degradation was assessed by measuring the diameter every 7th day of the experiment. Before use, scaffolds were disinfected by washing for 2 h in 70% Ethanol (Sigma Aldrich®, Switzerland) and thereafter, washing in Phosphate Buffered Saline (PBS) (Sigma Aldrich®, Switzerland).

### Perfusion bioreactor culture

The perfusion study was performed using a bidirectional perfusion flow system U-CUP (CELLEC BIOTEK AG, Switzerland). BMSCs cultured on scaffolds in static conditions and as 2D monolayers served as controls. Four million BMSCs re-suspended in 100 ul were seeded dropwise onto each scaffold in non-adherent 6-well plates and incubated for 1 h at 37°C before 5 ml of growth media per scaffold was added. After 24 h, scaffolds were transferred into the U-CUP bioreactor (Figures 1A,B). Due to holder dimensions and capacity, two scaffolds per system were cultured in a 10 ml volume of control medium [DMEM low glucose (Gibco®, Switzerland), 10% fetal calf serum (Gibco®, Switzerland), 50 μg/ml Primocin™ (InvivoGen, France)] vs. osteogenic culture medium [DMEM low glucose, 10% fetal calf serum, 50 μg/ml Primocin™, 5 mM Glycerol-2-Phosphate (Sigma Aldrich®, Switzerland), 50 μg/ml Ascorbic-Acid-2-Phosphate (Sigma Aldrich®, Switzerland), 10 nM Dexamethasone (Sigma Aldrich®, Switzerland)]. Oscillating flow in the U-CUP was created by the PHD Ultra™ syringe pump (Havard apparatus, United States). Perfusion velocity for the first 24 h was 400 μm/s, thereafter the velocity was reduced to 100 μm/s. Scaffolds in the static treatment group were transferred into new non-adherent 6-well tissue culture plates 24 h after seeding and each scaffold incubated in a 5 ml volume of control vs. osteogenic culture media. Media changes were performed 3× per week. Four experiments using a single BMSC donor per experiment were performed (*n* = 4). Samples per group and condition were obtained in triplicate. Samples for DNA quantification were taken at day 0, 7, and 21. RNA samples were taken at day 7 and 21. Histological analysis was performed at 21 days.

### Osteogenic differentiation in monolayer

BMSCs were seeded at a density of 20,000 cells/cm^2^ into a 24 well plate on Thermanox™ coverslips to prevent cell detachment and cultured in osteogenic vs. control media. Media was changed three times per week. At day 7 and 21 samples for DNA quantification and RNA were taken, per group and condition in triplicates. For Alizarin Red staining on day 21 BMSCs were washed twice with PBS before fixing with 10% formalin for 15 min. After washing alizarin red stain (40 mM; Sigma Aldrich®, Switzerland) was applied and incubated for 1 h on a horizontal shaker at room temperature (RT). Mineralization was assessed using a light microscope Microscope Axiovert 40 CFL (Carl Zeiss Microscopy GmbH, Germany).

### DNA quantification

DNA was quantified using Hoechst 33258 (Sigma Aldrich®, Switzerland). Scaffolds were digested in 1 ml Proteinase K (0,5 mg/ml in PBS containing 10.68 g/l NaH_2_PO_4_^*^ 2H_2_O, 8.45 g/l Na_2_HPO_4_^*^ 7H_2_O and 3.36 g/l Disodium-EDTA, pH 6.5; Roche Diagnostics GmbH, Germany) at 56°C overnight. The remaining scaffold was removed from the crude homogenate. DNA standards were prepared using calf thymus DNA (Invitrogen™, Switzerland). Standards and samples were diluted 1:5 with the assay solution containing 1 μg/ml Hoechst in PBS. After 15 min incubation in the dark the plate was read using a Perkin Elmer Viktor^3^ micro plate reader (Perkin Elmer, United States) at an excitation of 350 nm and an emission of 450 nm.

### Gene expression analysis

Three replicate samples per donor were separately processed and assessed for gene expression. For RNA isolation, samples were freeze thawed three times and lysed using a tissue lyser (QIAGEN, Switzerland) with stainless steel balls in TRI reagent (Molecular Research Center, USA). 10% 1-bromo-3-chloropropane (BCP) (Sigma Aldrich®, Switzerland) was added and the upper aqueous phase precipitated in 70% Ethanol. RNA was purified using RNeasy spin columns (QIAGEN, Switzerland) according to manufacturer's instructions. RNA was reverse transcribed into cDNA via a high capacity cDNA reverse transcription kit (Applied Biosystems, Switzerland). Real-time PCR was performed using Taqman reverse transcription reagent (life technologies™, Switzerland) and the QuantStudio™ 6 Flex Real-Time PCR System (life technologies™, Switzerland). For primer sequences, see Tables 1, 2. Relative fold change was calculated with the ddCT method. Normalization was performed to 18s rRNA and the average CT value of day 7 Thermanox™ control samples.

**Table 1:** Primer probe sequences used for real-time PCR with TaqMan method.

**Gene**	**Forward 5′-3′**	**Reverse 5′-3′**	**Probe 5′-3′**
Collagen I	5′-CCC TGG AAA GAA TGG AGA TGA T-3′	5′-ACT GAA ACC TCT GTG TCC CTT CA-3′	5′-CGG GCA ATC CTC GAG CAC CCT-3′
Osteocalcin	5′-AAG AGA CCC AGG CGC TAC CT-3′	5′-AAC TCG TCA CAG TCC GGA TTG-3′	5′-ATG GCT GGG AGC CCC AGT CCC-3′
Runx2	5′-AGC AAG GTT CAA CGA TCT GAG AT-3′	5′-TTT GTG AAG ACG GTT ATG GTC AA-3′	5′-TGA AAC TCT TGC CTC GTC CAC TCC G-3′
Collagen X	5′-ACG CTG AAC GAT ACC AAA TG-3′	5′-TGC TAT ACC TTT ACT CTT TAT GGT GTA-3′	5′-ACT ACC CAA CAC CAA GAC ACA GTT CTT CAT TCC-3′
E11	5′-GGT ACT CGC CCT AAA GAG CTG AA-3′	5′-GCA CAG AGT CAG AAA CGG TCT TTT-3′	5′-TTA CGC CCT GCT GCC AAC GTG C-3′
OPN	5′-CTC AGG CCA GTT GCA GCC-3′	5′-CAA AAG CAA ATC ACT GCA ATT CTC-3′	5′-AAA CGC CCA AGG AAA ACT CAC TAC C-3′

**Table 2:** Assay on demand used for real-time PCR with TaqMan method.

**Gene**	**Assay ID**
18 s	4310893E
ALP	Hs00758162_m1
Sox 9	Hs00165814_m1

### Histology

PLGA scaffolds were fixed in 70% methanol, dehydrated through an ascending series of ethanol and incubated overnight in a 1:1 mixture of Histo-Clear and embedded in paraffin (Sigma Aldrich®, Switzerland). Sections of 7 μm were taken using a Microm HM 355S microtome (Thermo Scientific, United States). Sections were dewaxed in Histo-Clear prior to histological staining. For Hematoxylin and Eosin (H&E), Mayer's hematoxylin (Fluka, Switzerland) was applied, blued in tap water then incubated in 1% Eosin (Fluka, Switzerland). Sections were dehydrated through graded ethanols, brought into xylene and mounted with Eukitt (O. Kindler GmbH & Co., Switzerland).

For von Kossa staining a 5% silver nitrate solution (Fluka, Switzerland) was applied and exposed to strong light. Following wash steps in 5% sodium thiosulfate and water, sections were counterstained in 0.1% nuclear fast red (Fluka, Switzerland). Sections were dehydrated through a series of graded alcohols, xylene and mounted with Eukitt. Imaging was performed using an Axioplan 2 Imaging microscope (Carl Zeiss Microscope GmBH, Germany) equipped with an Axiocam HRc (Carl Zeiss Microscope GmBH, Germany).

### Statistics

Data from individual donors and pooled from four donors (*n* = 4) are shown. Data for scaffold degradation and DNA content are presented as medians, error bars represent minimal and maximal data points. Gene expression data is presented from individual donors as the mean of three replicate samples ± standard deviations. Where appropriate statistical analysis was performed on data pooled from the four donors (*n* = 4). Statistical analysis was performed with GraphPad Prism 6 software (GraphPad Software Inc., La Jolla, CA, USA). Datasets were tested for normality using the D'Agostino-Pearson test for normality. As all data were non-normally distributed, the Kruskall-Wallis one-way analysis of variance test was used to test for differences between >2 groups. For the comparison between two groups, Mann-Whitney tests were performed. *p*-Value <0.05 was considered as statistically significant.

## Results

### Long term maintenance of BMSC-seeded PLGA scaffolds in a perfusion bioreactor

**Figure 2:**
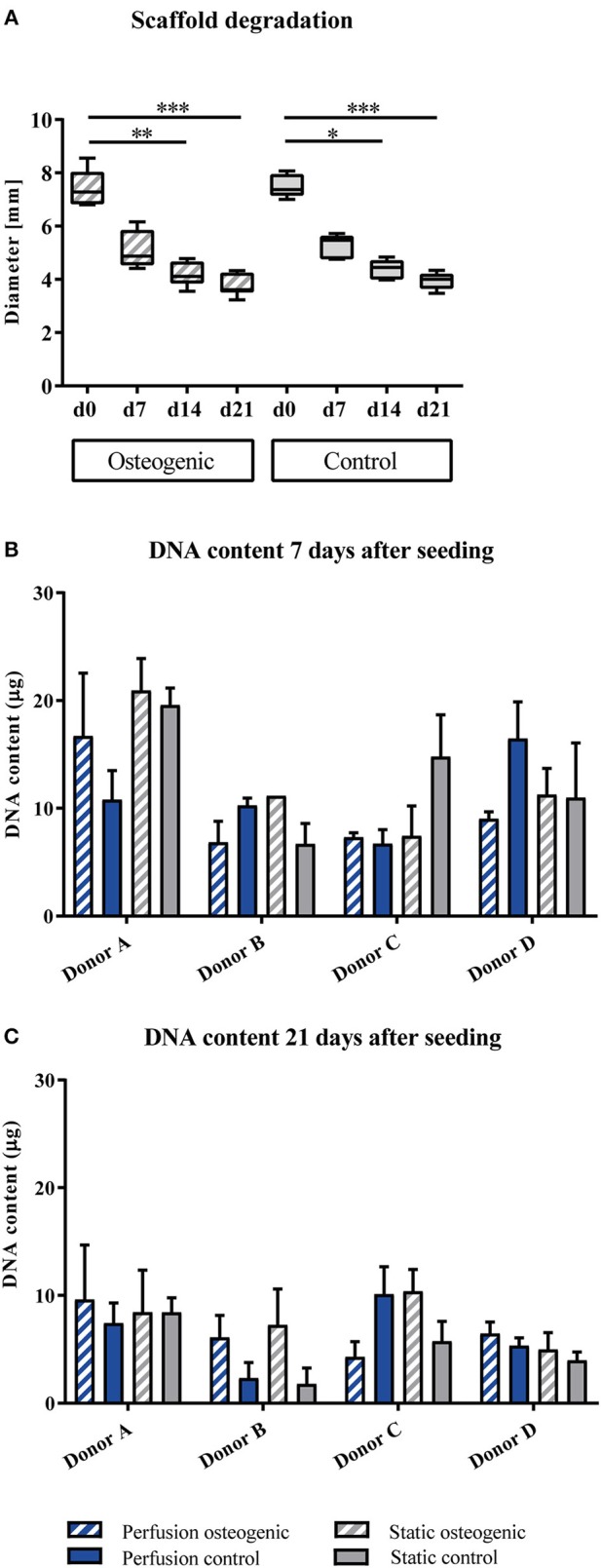
**(A)** Scaffold degradation. PLGA scaffolds containing BMSCs cultured in static conditions in osteogenic vs. control media as indicated were measured every 7th day of the experiment. Data presented are medians, error bars represent minimal and maximal data points, *n* = 6 scaffolds. **(B,C)** DNA quantification. BMSCs on PLGA scaffolds were cultured in static and perfusion conditions in osteogenic vs. control media as indicated. Samples taken after 7 and 21 days were analyzed using Hoechst. Data presented are means ± SD, *n* = 3 technical replicates per donor and experimental group. *P < 0.05, **P < 0.01, ***P < 0.001.

**Figure 3:**
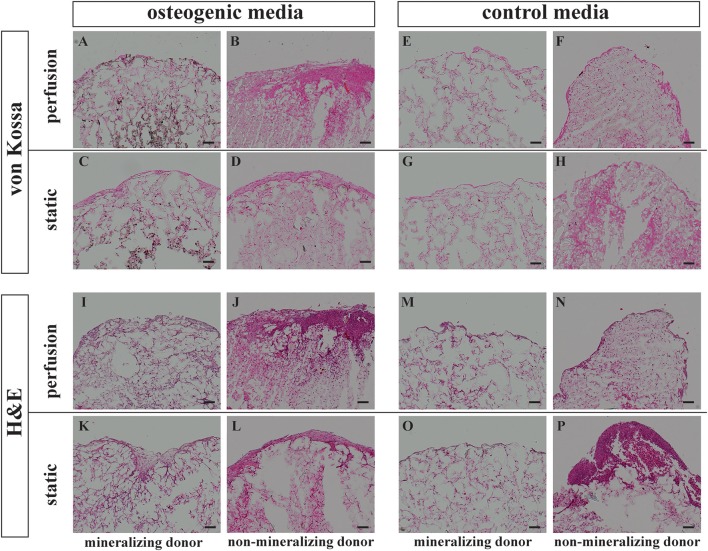
Histology von Kossa and Hematoxylin Eosin staining. BMSCs on PLGA scaffolds were cultured in perfusion conditions in osteogenic vs. control media as indicated. Samples were fixed at day 21 and paraffin embedded. Sections were stained with von Kossa or with Hematoxylin Eosin as indicated. Representative images of a mineralizing donor and a non-mineralizing donor were taken as indicated. Scale bar = 100 μm. Von Kossa staining of the perfusion osteogenic group from **(A)** mineralizing and **(B)** non-mineralizing donors. Von Kossa staining of the static osteogenic group from **(C)** mineralizing and **(D)** non-mineralizing donors. Von Kossa staining of the perfusion control group from **(E)** mineralizing and **(F)** non-mineralizing donors. Von Kossa staining of the static control group from **(G)** mineralizing and **(H)** non-mineralizing donors. H&E staining of the perfusion osteogenic group from **(I)** mineralizing and **(J)** non-mineralizing donors. H&E staining of the static osteogenic group from **(K)** mineralizing and **(L)** non-mineralizing donors. H&E staining of the perfusion control group from **(M)** mineralizing and **(N)** non-mineralizing donors. H&E staining of the static control group from **(O)** mineralizing and **(P)** non-mineralizing donors.

BMSCs were maintained in culture for 21 days on PLGA scaffolds in the perfusion bioreactor. PLGA gradually degraded throughout the culture period as shown by decreasing scaffold diameter (Figure 2A), which was accompanied by a reduction in total DNA content (Figures 2B,C). As scaffold degradation proceeded, differentiating BMSCs deposited an extracellular matrix such that by day 21 a significant quantity of mineralised extracellular matrix had accumulated within the scaffolds (Figures 3A,C).

### BMSCs synthesized a mineralised extracellular matrix within PLGA scaffolds

Representative images of extracellular matrix deposition from two donors are presented. H&E staining confirmed that independent of culture conditions, BMSCs were evenly distributed and viable for 21 days when cultured on PLGA scaffolds (Figures 3I–P). As indicated by Von Kossa staining, mineralization took place only in osteogenic media and was most prominent in the center of the scaffolds (Figures 3A,C). Mineralisation was generally more prominent in perfusion conditions (Figure 3A). This mineralisation pattern was observed in all donors except for one non-mineralizing donor which failed to mineralize on porous PLGA scaffolds under all culture conditions (Figures 3B,D,F,H).

**Figure 4:**
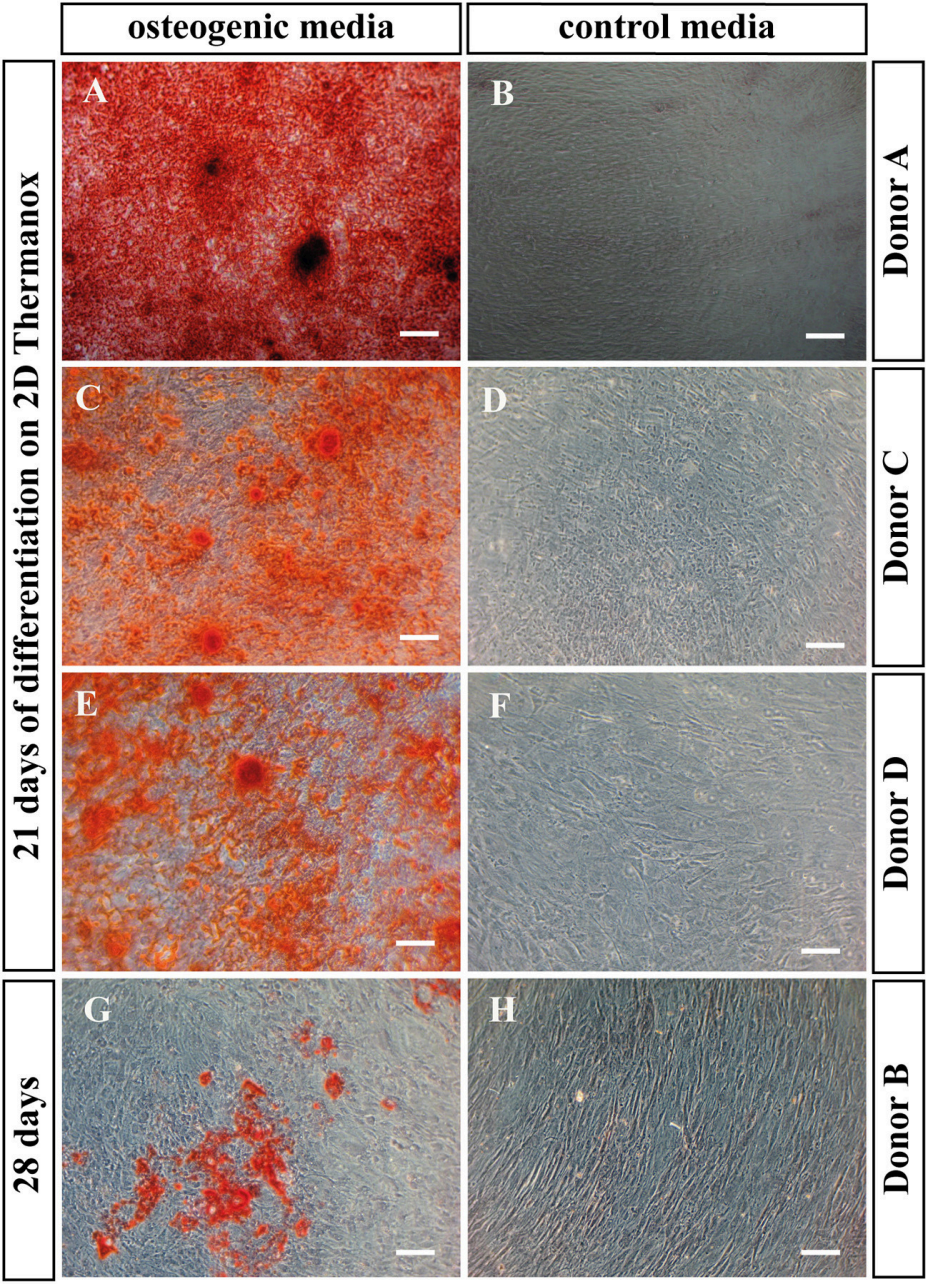
Alizarin red staining. BMSCs in monolayer on Thermanox™ cultured in osteogenic vs. control media as indicated. Cells were fixed and stained at day 21 except the non-mineralizing donor B, there the staining was performed at day 28, as after 21 days no mineralization was observed. Representative images of the osteogenic and the control group were taken. Scale bar = 100 μm. Donor A after 21 days of differentiation in **(A)** osteogenic vs. **(B)** control media. Donor C after 21 days of differentiation in **(C)** osteogenic vs. **(D)** control media. Donor C after 21 days of differentiation in **(E)** osteogenic vs. **(F)** control media. Donor B after 28 days of differentiation in **(G)** osteogenic vs. **(H)** control media.

Alizarin red staining was performed on BMSCs cultured on Thermanox™, to assess the ability of the cells to mineralize under osteogenic conditions in monolayer. All donors with the exception of the non-mineralizing donor mineralized after 21 days in osteogenic media (Figures 4A,C,E). This is in accordance with PLGA scaffold culture where the same donor also failed to mineralize. When BMSCs of the non-mineralizing donor were cultured for an additional week in osteogenic induction media on Thermanox™ (day 28) positive Alizarin red staining could be observed suggesting a slower response of this donor to osteogenic induction (Figure 4G). No mineralization was observed in controls of any BMSC donor (Figures 4B,D,F,H).

### PLGA scaffolds and perfusion culture enhanced the expression of osteogenic genes and collagen type X

**Figure 5:**
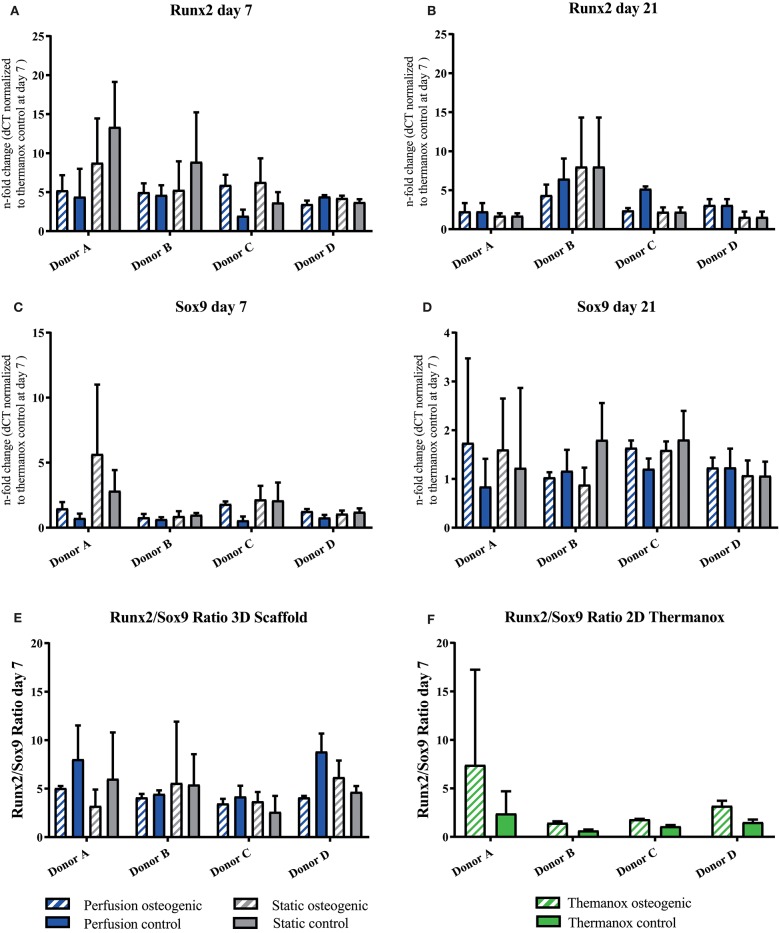
**(A–D)** Runx2 and Sox9 individual donors gene expression. BMSCs on PLGA scaffolds were cultured in static and perfusion conditions in osteogenic vs. control media as indicated. Samples taken after 7 and 21 days were analyzed using real time PCR. Relative fold change was calculated with the ddCT method. Normalization was performed to 18s rRNA and controls cultured on Thermanox™ at day 7. **(E)** Runx2/Sox9 ratio scaffold culture. BMSCs on PLGA scaffolds were cultured in static and perfusion conditions in osteogenic vs. control media as indicated. Samples taken after 7 days. Relative fold change calculated with the ddCT method of Runx2 at day 7 was divided by relative fold change of Sox9 at day 7. **(F)** Runx2/Sox9 ratio 2D culture. BMSCs in monolayer on Thermanox™ cultured in osteogenic vs. control media as indicated. Samples taken after 7 days. Relative fold change calculated with the ddCT method of Runx2 at day 7 was divided by relative fold change of Sox9 at day 7. Data presented are means ± SD, *n* = 3 technical replicates per donor and experimental group.

Porous PLGA scaffolds promoted osteogenic gene expression as shown by increases in fold change when compared to day 7 controls cultured on Thermanox™. The osteogenic transcription factor Runx2 was upregulated by BMSCs cultured on PLGA scaffolds in all conditions at day 7 and 21 (Figures 5A,B). Sox9 was robustly expressed by BMSCs throughout the culture period (Figures 5C,D). It has previously been shown that the Runx2/Sox9 ratio of BMSCs at day 7 is predictive of the cells mineralising potential at day 21 during 2D osteogenic differentiation (Loebel et al., 2015). Accordingly, in 2D conditions on Thermanox™ the Runx2/Sox9 ratio at day 7 was higher in osteogenic media compared to control media (Figure 5F). When cultured on PLGA scaffolds, BMSCs displayed a high Runx2/Sox9 gene expression ratio in both osteogenic and control media, supporting its osteoconductive properties (Figure 5E).

**Figure 6:**
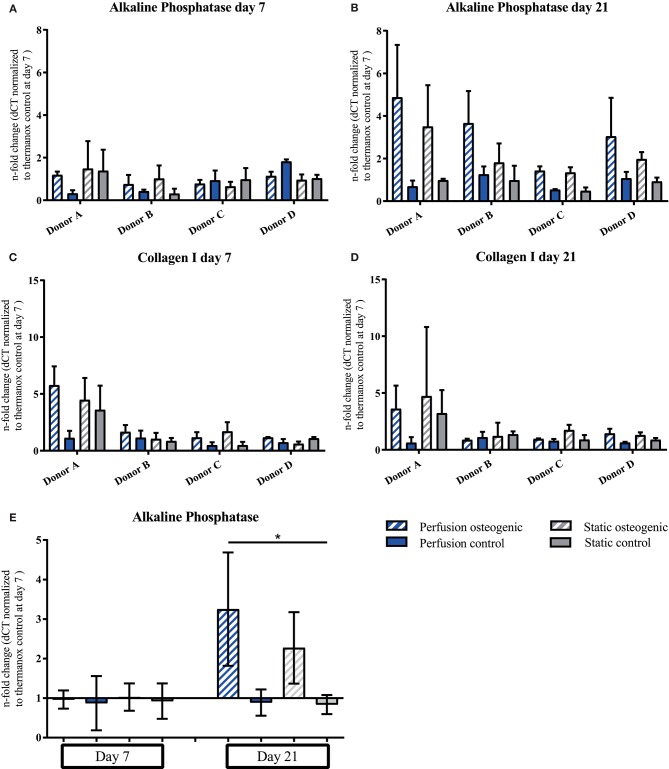
**(A–D)** Alkaline Phosphatase and Collagen I individual donors gene expression. BMSCs on PLGA scaffolds were cultured in static and perfusion conditions in osteogenic vs. control media as indicated. Samples taken after 7 and 21 days were analyzed using real time PCR. Relative fold change was calculated with the ddCT method. Normalization was performed to 18s rRNA and controls cultured on Thermanox™ at day 7. Data presented are means ± SD, *n* = 3 technical replicates per donor and experimental group where individual donors are presented. **(E)** Alkaline Phosphatase gene expression data pooled from the four individual donors **(A–D)**. Therefore, the three technical replicates per donor and group were averaged and the averaged values of each donor were pooled. Data presented are means ± SD, *n* = 4 donors, *P < 0.05.

Alkaline Phosphatase was expressed at day 7, increasing by day 21 in osteogenic groups (Figures 6A,B). Considering data pooled from all donors, Alkaline Phosphatase was significantly upregulated in the osteogenic perfusion group compared to the static control group at day 21 (perfusion osteogenic: 3.25 ± 1.43-fold change vs. static control: 0.84 ± 0.24-fold change, *p* = 0.0286; Figure 6E). Collagen I was expressed throughout the experiment with no significant difference between groups (Figures 6C,D).

**Figure 7:**
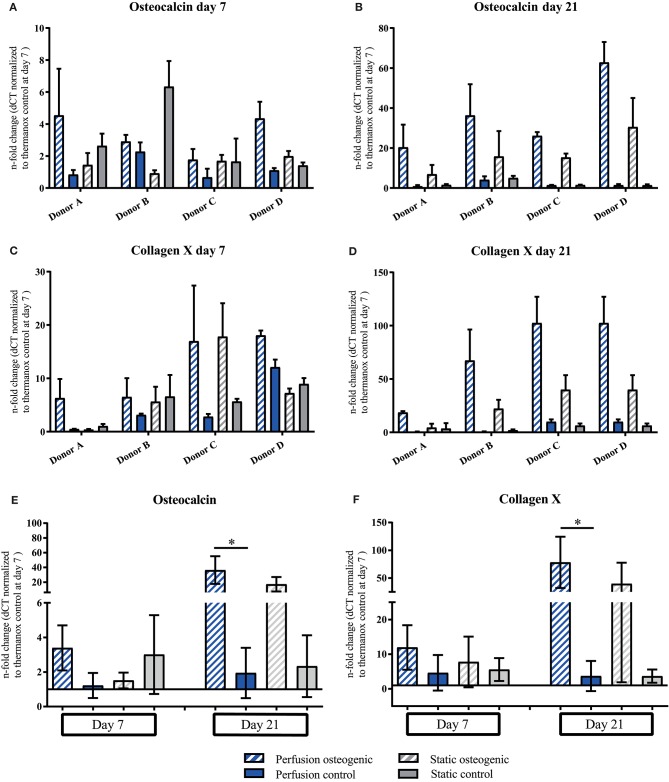
**(A–D)** Osteocalcin and Collagen X individual donors gene expression. BMSCs on PLGA scaffolds were cultured in static and perfusion conditions in osteogenic vs. control media as indicated. Samples taken after 7 and 21 days were analyzed using real time PCR. Relative fold change was calculated with the ddCT method. Normalization was performed to 18s rRNA and controls cultured on Thermanox™ at day 7. Data presented are means ± SD, *n* = 3 technical replicates per donor and experimental group where individual donors are presented. **(E,F)** Osteocalcin and Collagen X data pooled from the four individual donors **(A–D)**. Therefore, the three technical replicates per donor and group were averaged and the averaged values of each donor were pooled. Data presented are means ± SD, *n* = 4 donors, *P < 0.05.

Osteocalcin and Collagen X demonstrated similar gene expression patterns. PLGA scaffolds induced expression of Osteocalcin under all culture conditions at day 7 which increased in osteogenic perfusion groups in all donors by day 21 (Figures 7A,B). Considering data pooled from all donors, significant upregulation of Osteocalcin was observed in the osteogenic perfusion group compared to the control perfusion group at day 21 (Figure 7E; perfusion osteogenic: 36.37 ± 18.79-fold change vs. perfusion control: 1.94 ± 1.46-fold change, *p* = 0.014). Collagen X expression was upregulated in all groups at day 7 and was particularly high in the osteogenic perfusion groups in all donors at day 21 (Figures 7C,D). When data was pooled from all donors, significant upregulation of Collagen X was apparent in the osteogenic perfusion group compared to the control perfusion group at day 21 (Figure 7F; perfusion osteogenic: 78.17 ± 46.22-fold change vs. perfusion control 3.69 ± 4.36-fold change, *p* = 0.0451). A similar pattern but no statistically significant differences were observed between osteogenic static and control static groups (Figures 7E,F) for Osteocalcin and Collagen X pooled donor data.

**Figure 8:**
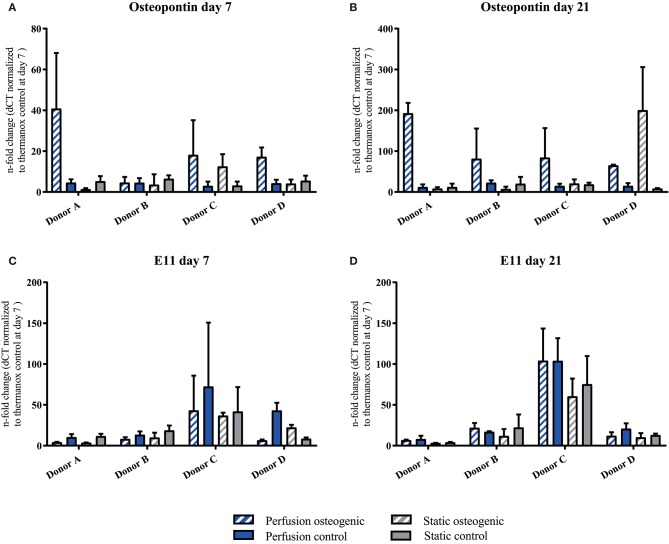
**(A–D)** Osteopontin and E11 individual donors gene expression BMSCs on PLGA scaffolds were cultured in static and perfusion conditions in osteogenic vs. control media as indicated. Samples taken after 7 and 21 days were analyzed using real time PCR. Relative fold change was calculated with the ddCT method. Normalization was performed to 18s rRNA and controls cultured on Thermanox™ at day 7. Data presented are means ± SD, *n* = 3 technical replicates per donor and experimental group.

Osteopontin was expressed at low levels at day 7 in all groups (Figure 8A). After 21 days Osteopontin was notably upregulated in the osteogenic perfusion group (Figure 8B). The early osteocyte marker E11 was upregulated in control and osteogenic media in all conditions where cells were cultured on porous PLGA scaffolds throughout the experiment (Figures 8C,D) No statistically significant differences in either Ell or Osteopontin gene expression between groups were observed when data from all donors were combined.

In summary, PLGA scaffold culture demonstrated osteoconductive properties, as even in control media under both static and perfusion conditions, osteogenic genes were upregulated. Perfusion culture further enhanced the expression of genes associated with a mature osteoblast phenotype in addition to Collagen X.

## Discussion

Our data show that porous PLGA scaffolds and perfusion conditions provided a favorable environment for the osteogenic differentiation of BMSCs supporting formation of a mineralised extracellular matrix. An interesting and novel finding of this work was that the 3D PLGA perfusion culture system enhanced the expression of Collagen type X—a gene typically associated with the hypertrophic chondrocyte phenotype, which was prominently expressed by cells cultured in the presence of osteogenic inductive factors (Figures 7C,D,F). Thus, the presented culture system appeared permissive for both direct and indirect routes of ossification that factor in the remodeling and healing processes occurring in native adult bone. Further, we demonstrate the suitability of this bioreactor system as a biomaterials testing platform to elucidate the important drivers of BMSC differentiation.

The U-CUP bioreactor successfully supported the long term culture of primary human BMSCs. This is in accordance with previous studies where this system was reported to maintain both freshly isolated and culture expanded marrow stromal cell populations (Wendt et al., 2003; Braccini et al., 2005; Papadimitropoulos et al., 2014). Cells cultured on 3D PLGA scaffolds demonstrated higher osteogenic gene expression when compared to 2D monolayer. The osteoconductive properties of the matrix provided by the biomaterial alone were sufficient to induce osteogenic differentiation, as shown by the upregulation of OPN and E11 in control media groups (Figures 8A–D). Osteogenic differentiation on 3D PLGA scaffolds was augmented further by treatment with osteogenic media under perfusion culture conditions. The late osteogenic markers Osteocalcin and Osteopontin which are known to be responsive to shear stress, were upregulated in BMSCs cultured in osteogenic media, while perfusion further increased the expression of these genes (Figures 7A,B,E, 8A,B; Kreke et al., 2005). The early osteocyte marker E11 was upregulated in all scaffold groups (Figures 8C,D). In bone, E11 is upregulated in response to fluid flow and shear stress and associated with increased dendrite number and length (Schulze et al., 1999; Zhang et al., 2006). Since E11 was upregulated in both static and perfusion groups, it suggests that the porous matrix provided by the PLGA scaffold was permissive for BMSC differentiation toward a mature osteoblast/early osteocyte phenotype. In the context of tissue engineering bone, the presence of OC, OPN and E11 is highly important to regulate appropriate mineralisation and tissue maturation. Nitric oxide (NO) and prostaglandin E_2_ (PGE_2_), which are known to be expressed by osteocytes and BMSCs in response to fluid flow and shear stress (Bakker et al., 2001) were measured by Elisa, but could not be detected (data not shown). This may have been due to the frequency of culture media replacement preventing accumulation up to detectable levels. Runx2 is a master regulator of BMSC osteogenic differentiation (Lefebvre and Smits, 2005). It was previously shown that a high Runx2/Sox9 ratio at day 7 of osteogenic differentiation is predictive of the osteogenic potential of BMSCs (Loebel et al., 2015). Data from the current study where BMSCs were osteogenically differentiated in monolayer corroborate the Runx2/Sox9 ratio as an osteogenic predictor (Figure 5F). However, the Runx2/Sox9 ratio at day 7 in cells cultured on porous PLGA scaffolds did not appear to be predictive for subsequent mineralization (Figure 5E). As both the PLGA scaffolds and perfusion culture enhanced the osteogenic response, the peak of the Runx2/Sox9 ratio may likely have occurred earlier than day 7. Runx2/Sox9 ratios were higher in scaffold controls compared to Thermanox™ controls, again supporting the osteoconductive properties of PLGA and the matrix architecture of the scaffolds.

Biomaterials may provide favorable conditions for either endochondral/intramembranous or indeed both ossification pathways. In light of this, we also assessed chondrogenic gene expression in our culture system, however we did not detect Collagen II nor aggrecan, again their expression might have peaked at an earlier time point. Additionally, glycosaminoglycans were not detected in histological nor biochemical analysis (data not shown). However, the 3D/PLGA perfusion culture system did induce the expression of Collagen X which was significantly upregulated in the osteogenic perfusion group at 21 days (Figure 7F). Collagen X is expressed by hypertrophic chondrocytes in the growth plate (Schmid and Linsenmayer, 1985) but also during fracture healing in the callus (Grant et al., 1987). It is suggested that Collagen X regulates not only the mineralization process (Bonen and Schmid, 1991; Kirsch and Wuthier, 1994) but also provides a suitable matrix for new bone formation. To our knowledge, assessment of Collagen X expression during the osteogenic differentiation of primary human BMSCs has not previously been reported. Collagen X, in addition to the expression of genes associated with direct osteogenesis suggests the presence of different cell phenotypes. We postulate that in the present system, whilst a proportion of BMSCs underwent direct osteogenic differentiation to mature osteoblasts, other BMSCs adopted a phenotype more akin to that of hypertrophic chondrocytes. Innate BMSC heterogeneity combined with variable micro-environmental conditions within the constructs could account for this. Differences in mass transport and fluid dynamics within porous scaffolds will invariably confer variable environmental conditions experienced by cells, which may predispose spatial differentiation gradients. The lineage pathways giving rise to osteoblasts and chondrocytes during physiological bone healing are not fully understood. Historically, it was believed hypertrophic chondrocytes in the cartilage anlagen undergo apoptosis prior to ossification occurring. However, recent studies present a new hypothesis that hypertrophic chondrocytes might be capable of trans-differentiation to osteoblasts (Bahney et al., 2014; Yang et al., 2014; Houben et al., 2016). The fact that our culture system allowed for a range of differentiated phenotypes to manifest, offers the potential to study the finer aspects of BMSC fate specification.

It is known that osteoblast and osteoprogenitor cell lines behave differently to primary human BMSCs which limits their suitability for biomaterials testing—particularly as we strive to develop autologous, clinical cell-based therapies (Czekanska et al., 2014). Therefore, we conducted our experiments with primary human BMSCs from a clinically relevant patient cohort. As in many studies assessing primary human BMSCs, we observed donor differences in measured outputs including gene expression. Donor variation is frequently apparent between primary BMSC cultures as they are a heterogeneous cell population derived from bone marrow in an unselected manner. Pooling BMSCs from different donors prior to an experiment may reduce variation, but also results in an average population that does not exist in reality (Stoddart et al., 2012). Thus, we assessed BMSC populations derived from individual donors in order to sample a biologically relevant population and maintain the clinical relevance of our findings. The variation in gene expression we observed is also partly attributable to the fact that whole construct analysis was performed which meant that potential spatial effects on cell behavior were not sufficiently represented. Hence, histological analysis is an indispensable tool to reveal spatial differences concerning cell behavior within a 3D scaffold and to address donor differences.

Three out of four donors formed a mineralized matrix under osteogenic perfusion conditions. The process of mineralization in biological tissues requires defined environmental conditions that are highly regulated. In the case of bone mineralization, extracellular matrix, namely collagen (Nudelman et al., 2010; Wang et al., 2012), non-collagenous bone proteins (Roach, 1994) and minerals are required. The ideal microenvironment for ossification appears to have been provided in the center of the scaffolds, as mineralization was predominantly observed there. A similar process occurs in long bone development, where in the center of the cartilage anlagen mineralizes to create the primary ossification center (Gilbert, 2016). It may be that non-collagenous proteins might be better retained in the scaffold center, providing optimal conditions for mineralization. The non-mineralizing donor, which did not mineralize in 3D culture on PLGA also displayed very low and delayed mineralization by standard monolayer osteogenesis assay (Figures 3B,D,F,H, 4G). As this donor mineralized less *in vitro* compared to the other three donors, it may be that the original bone marrow sample contained a greater proportion of uncommitted BMSCs vs. osteoprogenitors/BMSCs with a high mineralizing capacity. At the gene expression level, the non-mineralizing donor was not outstanding in terms of less upregulated osteogenic genes. This discrepancy between what is observed at gene expressional vs. protein level, highlights the importance of investigating cellular differentiation using different methods including those which assess mineral and extracellular matrix production directly.

Biomaterials designed for bone regeneration may perform well when tested *in vitro* under static conditions, however may subsequently fail when tested in fracture models (Hulsart-Billström et al., 2016). This study addressed the unmet need to develop a more physiologically relevant testing platform by using primary human cells in a 3D environment and under perfused conditions.

During human movement bone cells experience a complex biomechanical environment. In addition to stresses and strains interstitial fluid flow through the lacunar-canalicular spaces generates shear stress. Our flow velocity of 100 μm/s was close to the interstitial fluid flow velocity in human cortical bone (100 μm/s; Kufahl and Saha, 1990) and mice (80 μm/s; Zhou et al., 2008). Fluid induced shear stresses range in the human bone from 0 to 20 dynes/cm^2^ depending on the cellular location (Mi et al., 2005a,b). However, as our porous scaffold contained highly irregular geometries non-uniform flow patterns could be expected inside the scaffold. Thus, shear stress variations greater than one order of magnitude could be expected even when constant flow velocities are applied (Boschetti et al., 2006; Jungreuthmayer et al., 2009). In addition to the biochemistry and the mechanical environment of a scaffold, matrix architecture plays a tremendous role in driving cellular differentiation (Melchels et al., 2010; Saito et al., 2013). In the present study, several characteristics may have contributed to the osteoinductive properties of the PLGA scaffolds including pore size. The pore size of our salt-leached scaffolds ranged between 100 and 150 μm which is similar to the pore size of the Haversian canals in human cortical bone (Wang and Ni, 2003). The suitability of this pore size for bone ingrowth was previously determined in 1971 by Klawitter and Hulbert (1971) and has been successfully confirmed by others with the use of micro-CT (Jones et al., 2007). There are other features of the scaffolds that may have influenced BMSC differentiation including surface topography and stiffness. BMSCs cultured in our 3D culture system would have experienced different stiffnesses depending upon their location. For example cells adhered directly to the biomaterial vs. cells encapsulated in extracellular matrix within a scaffold pore. A stiff substrate is known to direct BMSCs toward osteogenic differentiation (Engler et al., 2006). This has the advantage that the process is faster in contrary to natural healing process of long bones whereby a cartilage intermediate is formed initially. However, stiff biomaterials often fail *in vivo*, as fast matrix deposition combined with a lack of vascularization limits nutrient and gas exchange (Nomi et al., 2002; Ko et al., 2007; Thompson et al., 2015). Therefore, attention has turned toward softer substrates and creating an environment favorable for chondrogenesis (Dennis et al., 2015; Thompson et al., 2015). The endochondral ossification route is a lengthier process, but chondrocytes are larger compared to osteoblasts and thus the fracture gap is bridged quicker. Further, the endochondral ossification has the advantage that hypertrophic chondrocytes are known to secrete a wide range of soluble osteogenic and angiogenic factors thus promoting vascular invasion of the tissue (Mackie et al., 2011). Our culture system appeared permissive in supporting BMSC differentiation pathways involved in both intramembranous and endochondral ossification. A tissue engineered construct combining the advantages of both ossification routes could be very favorable in mediating rapid and functional bone regeneration.

How BMSC-seeded PLGA scaffolds would perform *in vivo* requires further investigation. It is desirable that materials implanted into bone defects degrade at a suitable rate to allow new matrix deposition, mineralisation and vascular ingrowth to take place. The degradation of PLGA in this study supports previous observations using these scaffolds (Bardsley et al., 2016). PLGA degradation rate may be modified by adjusting internal factors; ratio of glycolic vs. lactic portion, porosity, size etc (Lu et al., 1999; Wu and Ding, 2005; Makadia and Siegel, 2011) and external factors; local pH, mechanical loading, temperature (Middleton and Tipton, 2000; Grayson et al., 2005; Yang et al., 2010). Ultimately, the success of a tissue engineering strategy for bone repair require testing using an appropriate pre-clinical model. Factors, such as mechanical loading, paracrine signaling between cell types, the periosteum and the immune system all play a tremendous role in fracture healing (Dwek, 2010; Marsell and Einhorn, 2011).

In conclusion, we report the novel use of a perfusion bioreactor for the osteogenic differentiation of primary human BMSCs on porous PLGA scaffolds. Our 3D culture system supported long term culture and promoted osteogenesis via both direct and indirect ossification routes. The design features of the bioreactor offer the possibility to screen multiple biomaterials in parallel and under controlled conditions. The opportunity to investigate the finer aspects of BMSC differentiation under more physiological conditions may improve our understanding of the micro-environmental cues governing BMSC fate. This may in turn help us to understand the causes of non-union fracture, identify mechanistic targets and enable the fine-tuning of biomaterial design in order to deliver reliable tissue engineered approaches for bone repair.

## Author contributions

All authors have approved the final version of the manuscript and agree to be accountable for all aspects of the work in ensuring that questions related to the accuracy or integrity of any part of the work are appropriately investigated and resolved. The authors contributed to the work as follows: CM and JB, design and conception of work, acquisition, analysis and interpretation of data, drafting the article, revision; KB, production of PLGA scaffolds; AE, MA, and MS, design and conception of work, interpretation of data, revision.

### Conflict of interest statement

The authors declare that the research was conducted in the absence of any commercial or financial relationships that could be construed as a potential conflict of interest.
